# Post-partum Coronavirus Disease 19 Like Pneumonia Before the COVID-19 Italian Pandemic Outbreak: A Case Report

**DOI:** 10.3389/fmed.2021.576865

**Published:** 2021-05-11

**Authors:** Andrea Busnelli, Alessandro Bulfoni, Andrea Caprotti, Stefano Acerboni, Paolo Emanuele Levi-Setti

**Affiliations:** ^1^Department of Biomedical Sciences, Humanitas University, Milan, Italy; ^2^Division of Gynecology and Reproductive Medicine, Department of Gynecology, Fertility Center, IRCCS Humanitas Research Hospital, Milan, Italy; ^3^Division of Obstetrics and Gynecology, IRCCS Humanitas Research Hospital, Humanitas S. Pio X Hospital, Milan, Italy; ^4^Division of Radiology, IRCCS Humanitas Research Hospital, Humanitas S. Pio X Hospital, Milan, Italy

**Keywords:** COVID-19, coronavirus, pneumonia, *Klebsiella pneumoniae*, post-partum

## Abstract

Here we present a case of severe post-partum pneumonia that we observed at the end of January 2020. Specimen of blood was cultured and revealed *Klebsiella pneumoniae* bacteremia. However, the course of infection was atypical and the recovery time particularly long. Subsequently emerged COVID-19 hallmarks suggested to re-evaluate the case. After a multidisciplinary consultation, we concluded that, considering the clinical and imaging characteristics, the most likely hypothesis was that the patient was affected by novel Coronavirus pneumonia. The present case supports the hypothesis that Coronavirus might have circulated in northern Italy for weeks before its official detection.

## Introduction

Coronavirus disease 2019 (COVID-19), caused by the severe acute respiratory syndrome Coronavirus 2 (SARS-CoV-2), rapidly escalated to a pandemic in the span of 2 months and has compromised healthcare systems around the world (1). The virus was first confirmed to have spread to Italy on 31 January 2020, when two Chinese tourists in Rome tested positive for the virus. On February 21, 2019, the first Italian patient with Coronavirus COVID-19 was diagnosed, a 38-year-old man hospitalized at Codogno Hospital, Lodi, in northern Italy. Also, in northern Italy, on February 21, 2020, another outbreak of viruses was discovered in Vò Euganeo (Padua) and, in the Veneto region, the first death was reported, a 78-year-old man in a hospital in Padua. He was the first of a long series of deaths (2). As of 6 June 2020, Italy has 35,877 active cases, one of the highest in the world. Overall, there have been 234,801 confirmed cases and 33,846 deaths (a rate of 561 deaths per million population), while there have been 165,078 recoveries or dismissals. By 5 June, Italy had tested about 2,565,000 people. Due to the limited number of tests performed, the real number of infected people in Italy, as in other countries, is estimated to be higher than the official count (3).

Available epidemiological models failed to justify such a rapid growth in the number of infections. Still undemonstrated theories sustain that the new Coronavirus may have circulated in northern Italy for weeks before it was detected, seriously complicating efforts to track and control its rapid spread across Europe. Preliminary evidence suggested the virus could have been spreading below the radar in the quarantined areas. The real beginnings of the outbreak, which has spread from Italy across Europe, were probably seeded at least two or 3 weeks before the first detection and possibly before flights between Italy and China were suspended at the end of January.

## Case Report

A 38-year-old primiparous woman was admitted to the Obstetrics and Gynecology Unit of the Humanitas S. Pio X Hospital in Milan (Italy) at 38 weeks 4 days of gestation because of oligohydramnios [Amniotic Fluid Index (AFI = 3 cm)] on January 23rd, 2020. The fetus was in cephalic presentation, with a heart rate of 134 beats per minute. The placenta was positioned anteriorly, the cervical length was 21 mm, and the umbilical artery appeared normal on Doppler examination.

During the previous 1.5 years, the patient had been well but unable to conceive. The patient was referred to an expert fertility consultant (PL-S), Director of the Humanitas Fertility Center in Rozzano (Milan, Italy). Transvaginal ultrasound showed bilateral ovarian endometrioma. Ovarian reserve resulted depleted [Antral follicle count (AFC) = 6; Anti-Müllerian Hormone (AMH) < 0.1 ng/ml]. She completed two cycles of *in vitro* fertilization (IVF); two high-quality embryos were transferred during each cycle. The second IVF attempt resulted in the present singleton pregnancy.

She was otherwise healthy and was a non-smoker taking no medications. After the admission, the patient was hydrated. Nevertheless, the ultrasound reevaluation confirmed oligohydramnios and the labor was induced with intravaginal prostaglandin E2. During labor, the cardiotocography tracing was characterized by reduced variability and showed repetitive variable decelerations suggesting a high probability of fetal hypoxia/acidosis according to the International Federation of Gynecology and Obstetrics (FIGO) consensus guidelines on intrapartum fetal monitoring (4). An emergency cesarean section was promptly carried out under combined spinal-epidural anesthesia on January 24th, 2020. Cefazolin was administered intravenously before incision of the skin. The infant was delivered 5 min after the skin incision. The 1 and 5-min scores were 8 and 10, respectively. The umbilical cord blood parameters were reassuring and didn't reflect a fetal hypoxic stress. Cesarean section was exempt from surgical complications.

**Figure 1:**
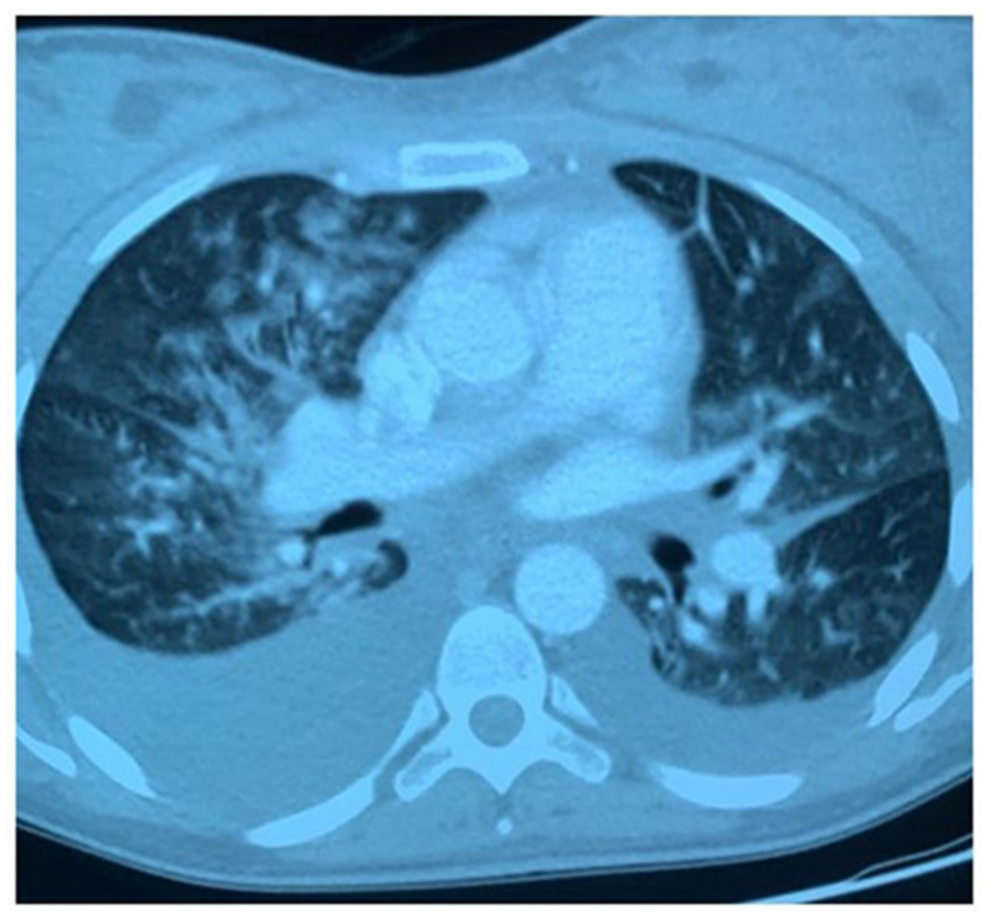
Pulmonary computed-tomographic (CT) performed on January 26th, 2020: bilateral confluent and patchy ground-glass and consolidative pulmonary opacities, small, bilateral pleural effusions.

Twenty hours after the c-section, mild dyspnea on exertion developed, associated with slight leg edema. On examination, the blood pressure was 135/72 mm Hg, the respiratory rate 16 breaths per minute, and the oxygen saturation 99% while the patient was breathing ambient air; the temperature was 37.6°C. An electrocardiogram (ECG) showed sinus rhythm at 118 beats per minute. Blood levels of total bilirubin, total protein, albumin, calcium, alanine aminotransferase, and aspartate aminotransferase were normal, as were tests of renal function; other test results are shown in Table 1. She was given broad spectrum antibiotics and strictly monitored. The day after (50 h after the c-section) dyspnea progressively worsened and a productive cough appeared. Body temperature was 38.2°C, the respiratory rate 16 breaths per minute, and the oxygen saturation 97% while the patient was breathing ambient air. Lung auscultation revealed bi-basal rhonchi and vescicular sounds bibasaly reduced. After a consultation with a pulmonologist, pulmonary computed-tomographic (CT) was planned and showed bilateral confluent and patchy ground-glass and consolidative pulmonary opacities, small, bilateral pleural effusions and no evidence of pulmonary embolism, aortic aneurysm, or pericardial effusion (Figure 1). Specimen of blood was cultured and revealed *Klebsiella pneumoniae* bacteremia. An infectious disease specialist consultation was required and a targeted antibiotic therapy with Metronidazole and Ceftadizime was started. After 4 days, the clinical picture showed no significant improvements. A new pulmonary CT was performed and confirmed the absence of benefits of the current treatment. The infectious disease specialist therefore decided to add the administration of Meropenem to the ongoing antibiotic therapy.

**Table 1:** Blood test performed on January 25th, 2020.

**Variable**	**Value**	**Reference range**
White blood cells (×10^9^/L)	16.61	3.50–10.50
Red blood cells (×10^12^/L)	3.26	3.90–5.00
Hemoglobin (g/dl)	10.8	12.0–15.5
Hematocrit (%)	30.3	34.9–44.5
Mean corpuscolar volume (fl)	92.9	80–99
Mean cell hemoglobin (pg)	33.1	27–32
Mean corpuscular hemoglobin concentration (g Hb/dL RBC)	35.6	32.0–36.0
Platelets (×10^9^/L)	145	130–400
Neutrophils (×10^9^/L)	15.26	1.50–8.00
Eosinophils (×10^9^/L)	0	0.00–0.50
Basophils (×10^9^/L)	0.03	0.00–0.20
Lymphocites (×10^9^/L)	0.65	0.70–5.00
Monocytes (×10^9^/L)	0.67	0.10–1.00

**Figure 2:**
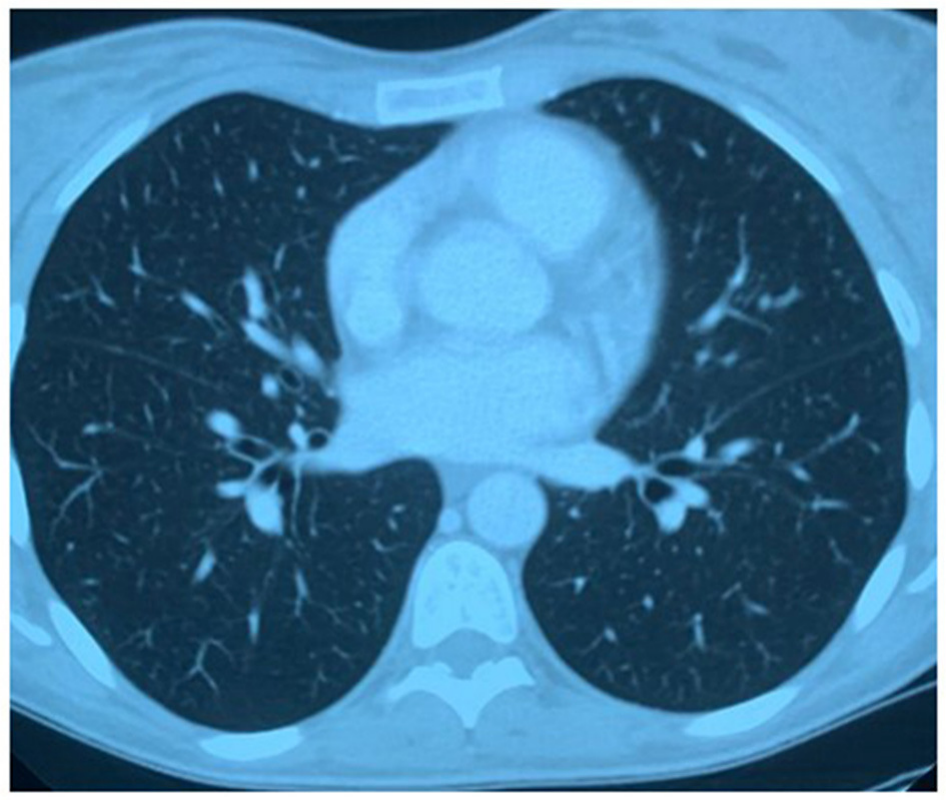
Pulmonary computed-tomographic (CT) performed on February 13th, 2020: complete restoration of the physiological transparency of the lung fields without residual consolidation thickenings and thin bilateral basal fibrotic outcomes.

The clinical picture and the laboratory findings slowly and progressively improved. Pulmonary CT was repeated 20 days after the first day of hospitalization and demonstrated complete restoration of the physiological transparency of the lung fields without residual consolidation thickenings and thin bilateral basal fibrotic outcomes (Figure 2). The patient was discharged in good health after 21 days of hospitalization. Follow-up appointments were scheduled: physical examinations and laboratory tests demonstrated a complete resolution of the clinical picture.

## Discussion

Herein, we presented a case of severe post-partum pneumonia before the COVID-19 Italian pandemic outbreak. The responsible agent was considered *K. pneumoniae*. However, the evidence that subsequently emerged about the clinical and imaging features of women affected by COVID-19 suggested to reassess the case. After a multidisciplinary consultation, we concluded that the most likely hypothesis was that the patient was affected by novel Coronavirus pneumonia. Co-infection with *K. pneumoniae* probably inhibited the host immune system making the recovery process particularly prolonged.

The arguments in support of our thesis are: (1) the patient clinical characteristics compatible with those of COVID-19; (2) unresponsiveness to targeted antibiotic therapy; (3) CT findings: chest CT is considered the imaging method of choice in the diagnosis of COVID-19 infection; CT characteristics of the present case are consistent with the hallmarks of COVID-19 infection (5–7); (4) the imaging aspect is not the most typical for *K. pneumoniae*.

On the other hand, some clinical features that might question our interpretation must be disclosed. First of all, the observed lung insemination and bilateral pleurisy in the presence of clinical signs of fever, cough, and dyspnea could suggest a bacterial etiology. The hematogenous dissemination of *K. pneumoniae* supports this hypothesis. However, also a nosocomial gram negative bacterium infection cannot be excluded. Second, in COVID-19 infection, bilateral pleurisy is rare and ground grass infiltrates are predominantly sub-pleural. Finally, ceftazidime resistance could also appear for *K. pneumoniae* and metronidazole would have not affected gram-negative bacteria.

In the absence of the real time reverse transcription–polymerase chain reaction (real time RT–PCR) test for SARS-CoV-2 and the Immunoglobulin G (Ig G) virus-specific antibody detection for COVID-19, the certainty in diagnosis is obviously unattainable. However, the low level of accuracy of such tests that was feared in the early phase of the pandemic and the uncertainties about how long people who recovered would have had immunity dissuaded us from contacting the patient for confirmation tests. Furthermore, even if the serological tests were positive, it could not have been excluded that the patient had contracted the infection later without developing significant symptoms.

In conclusion, here we presented a case highly suspected for COVID-19 observed at the end of January 2020 supporting the hypothesis of a Coronavirus Italian spreading before the official outbreak.

## Data Availability Statement

The raw data supporting the conclusions of this article will be made available by the authors, without undue reservation.

## Ethics Statement

Ethical review and approval was not required for the study on human participants in accordance with the local legislation and institutional requirements. The patient/participant of this study provided her written informed consent for the analysis and publication of any her potentially identifiable images or data.

## Author Contributions

PL-S conceived the manuscript. ABus, ABul, SA, and AC collected data. ABus wrote the first draft. All authors revised the manuscript and approved the final version.

## Conflict of Interest

The authors declare that the research was conducted in the absence of any commercial or financial relationships that could be construed as a potential conflict of interest.
